# Salicylic Acid Priming Improves Cotton Seedling Heat Tolerance through Photosynthetic Pigment Preservation, Enhanced Antioxidant Activity, and Osmoprotectant Levels

**DOI:** 10.3390/plants13121639

**Published:** 2024-06-14

**Authors:** Ashim Kumar Das, Protik Kumar Ghosh, Sheikh Arafat Islam Nihad, Sharmin Sultana, Sanjida Sultana Keya, Md. Abiar Rahman, Totan Kumar Ghosh, Munny Akter, Mehedi Hasan, Umme Salma, Md. Mahadi Hasan, Md. Mezanur Rahman

**Affiliations:** 1 Department of Agroforestry and Environment, Bangabandhu Sheikh Mujibur Rahman Agricultural University, Gazipur 1706, Bangladesh; ashimbsmrau@gmail.com (A.K.D.); abiar@bsmrau.edu.bd (M.A.R.); 2 Department of Agronomy, Bangabandhu Sheikh Mujibur Rahman Agricultural University, Gazipur 1706, Bangladesh; protikbsmrau@gmail.com (P.K.G.); munny@bsmrau.edu.bd (M.A.); 3 Plant Pathology Division, Bangladesh Rice Research Institute, Gazipur 1701, Bangladesh; nihad402@gmail.com; 4 Institute of Biotechnology and Genetic Engineering, Bangabandhu Sheikh Mujibur Rahman Agricultural University, Gazipur 1706, Bangladesh; sharminomi87@gmail.com; 5 Institute of Genomics for Crop Abiotic Stress Tolerance, Department of Plant and Soil Science, Texas Tech University, Lubbock, TX 79409, USA; skeya@ttu.edu; 6 Department of Crop Botany, Bangabandhu Sheikh Mujibur Rahman Agricultural University, Gazipur 1706, Bangladesh; totan@bsmrau.edu.bd; 7 Department of Agriculture, Bangabandhu Sheikh Mujibur Rahman Science and Technology University, Gopalganj 8100, Bangladesh; mhjackey26@gmail.com; 8 Department of Biochemistry and Molecular Biology, Primeasia University, Dhaka 1213, Bangladesh; ummesalma.jubmb15@gmail.com; 9 State Key Laboratory of Grassland Agro-Ecosystems, College of Ecology, Lanzhou University, Lanzhou 730000, China

**Keywords:** antioxidant enzymes, osmotic balance, photosynthetic pigments, reactive oxygen species, salicylic acid

## Abstract

The escalating global temperatures associated with climate change are detrimental to plant growth and development, leading to significant reductions in crop yields worldwide. Our research demonstrates that salicylic acid (SA), a phytohormone known for its growth-promoting properties, is crucial in enhancing heat tolerance in cotton (*Gossypium hirsutum*). This enhancement is achieved through modifications in various biochemical, physiological, and growth parameters. Under heat stress, cotton plants typically show significant growth disturbances, including leaf wilting, stunted growth, and reduced biomass. However, priming cotton plants with 1 mM SA significantly mitigated these adverse effects, evidenced by increases in shoot dry mass, leaf-water content, and chlorophyll concentrations in the heat-stressed plants. Heat stress also prompted an increase in hydrogen peroxide levels—a key reactive oxygen species—resulting in heightened electrolyte leakage and elevated malondialdehyde concentrations, which indicate severe impacts on cellular membrane integrity and oxidative stress. Remarkably, SA treatment significantly reduced these oxidative stresses by enhancing the activities of critical antioxidant enzymes, such as catalase, glutathione *S*-transferase, and ascorbate peroxidase. Additionally, the elevated levels of total soluble sugars in SA-treated plants enhanced osmotic regulation under heat stress. Overall, our findings reveal that SA-triggered protective mechanisms not only preserve photosynthetic pigments but also ameliorate oxidative stress and boost plant resilience in the face of elevated temperatures. In conclusion, the application of 1 mM SA is highly effective in enhancing heat tolerance in cotton and is recommended for field trials before being commercially used to improve crop resilience under increasing global temperatures.

## 1. Introduction

The marked increase in both frequency and severity of abiotic stresses—including heat waves, cold snaps, droughts, and floods—substantially compromises crop yields and threatens the sustainability of agriculture [1,2]. Global warming, characterized by elevated environmental temperatures, is predicted to intensify these challenges, with more severe and frequent heat stress episodes anticipated in the future [3,4,5]. Heat stress impairs plant performance, adversely affecting growth, photosynthesis, mineral uptake, and yield [6,7,8,9]. In addition, heat stress disrupts cellular homeostasis, leading to an overwhelming upsurge in reactive oxygen species (ROS). This results in a cascade of oxidative burden, which is characterized by an excessive accumulation of hydrogen peroxide (H_2_O_2_), which in turn leads to an increase in malondialdehyde (MDA) production [10,11]. Indeed, plants have evolved sophisticated physiological, biochemical, cellular, and molecular regulatory mechanisms to meticulously orchestrate their responses to heat stress [12]. The mechanisms involved reduced water loss, maintaining the integrity of the cell membrane, scavenging ROS, and strengthening both enzymatic and non-enzymatic antioxidant defense systems [13,14]. Despite these adaptive mechanisms, the rising global temperature and erratic climatic patterns exacerbate heat stress impact on a wide range of crops, including cotton (*Gossypium* spp.), highlighting the urgent need for innovative strategies to improve plant resilience [3]. 

Cotton has been considered the preeminent plant for natural fibers, accounting for 35% of the global market share, making it an indispensable commercial crop worldwide [15]. *Gossypium hirsutum*, the primary species of cotton, is grown in over 95% of the world’s cotton-producing areas, supplying vital natural fibers for the textile industry and yielding important by-products, including biofuels, food products, and edible oils [16,17,18]. Although cotton plants can withstand temperatures up to 43 °C without apparent adverse effects when soil moisture is adequate, prolonged heat stress significantly impairs their growth, development, yield, and the quality of the fiber produced [18,19,20]. Decades-long endeavors have been devoted to breeding and utilizing biotechnological strategies to develop cotton varieties with enhanced heat tolerance. Despite these efforts, achieving significant progress remains a formidable challenge, necessitating substantial investment of both time and resources [21]. Recently, the application of phytohormones, such as salicylic acid (SA), has gained scientific attention as a means of alternative and promising strategy for enhancing resilience against multiple abiotic stresses [22,23,24,25]. SA is recognized for its crucial role in modulating a multitude of key physiological and biochemical processes, including optimizing the acquisition of nutrients, enhancing the retention of water, coordinating the closure of stomata, stimulating the activity of photosynthesis, accumulating osmolytes, and detonating the activation of antioxidant defense mechanisms. These mechanisms endow plants with an extraordinary ability to withstand abiotic stresses, including the effect of scorching heat [26,27,28]. A plethora of research has demonstrated that applications of SA have improved the drought tolerance in canola (*Brassica napus* L.) [29], heat tolerance in maize (*Zea mays*) [30], cold stress in wheat (*Triticum aestivum*) [31], cadmium stress in rice (*Oryza sativa*) [32], and salinity stress in sorghum (*Sorghum bicolor*) [33].

Given the myriad benefits of SA, we anticipated that the supplementation of SA could play a crucial role in nullifying the pernicious consequences of heat stress on cotton plants. This intervention is expected to regulate a multitude of mechanisms associated with morphological, physiological, and biochemical features. Considering this, the present study delved into the pivotal functions of SA in alleviating heat stress-induced damage in the CB-12 cotton variety, a widely acclaimed upland cotton cultivar extensively grown in the cotton-rich regions of Bangladesh. Our study focused on a thorough investigation of diverse morpho-physiological and biochemical parameters. These encompassed a comprehensive assessment of (i) plant growth characteristics and subsequent biomass accumulation, (ii) abundance of diverse photosynthetic pigments, (iii) heat-induced H_2_O_2_ accumulation and their consequential impact on membrane lipid peroxidation and electrolyte leakages in leaves, (iv) levels and/or activities of essential non-enzymatic and enzymatic antioxidants, and (v) accumulation of various osmoprotectants, and their remarkable role in maintaining plant water status. 

## 2. Results

### 2.1. Salicylic Acid Positively Modulated the Growth and Phenotype of Heat-Stressed Cotton Plants

**Figure 1:**
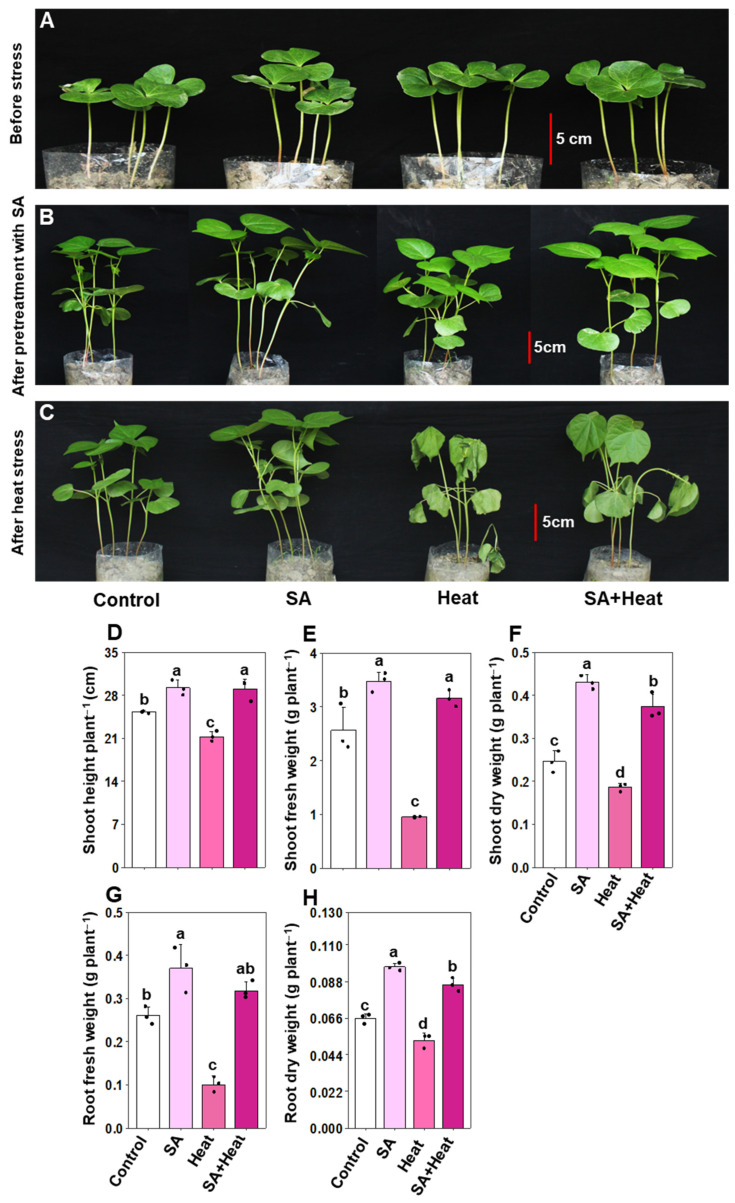
Impact of exogenously applied salicylic acid (SA, 1 mM) on growth-related characteristics of cotton plants subjected to 48 h of heat stress. Representative images of cotton plants captured at different stages: (**A**) before priming with 1 mM SA (pretreatment), (**B**) after 10 days of 1 mM SA priming (pre-stress), and (**C**) following exposure to 48 h of heat stress. Growth parameters assessed include (**D**) shoot height per plant, (**E**) shoot fresh weight per plant, (**F**) shoot dry weight per plant, (**G**) root fresh weight per plant, and (**H**) root dry weight per plant under different treatments. Data are presented as mean values with standard deviations (*n* = 3). Statistically significant treatment differences, as assessed by the least significant difference (LSD) test (*p* < 0.05), are indicated by distinct letters above the bars. Treatments include water-treated non-stressed plants (Control), 1 mM SA-primed non-stressed plants (SA), water-treated heat-exposed plants (Heat), and 1 mM SA-primed heat-exposed plants (SA+Heat).

To assess the effects of SA and water pretreatment on cotton plants subjected to heat stress, we evaluated various growth features along with the phenotypic appearance (Figure 1A–F). Phenotypic inspections revealed that heat stress led to significant alterations, such as leaf wilting and shrinking in plants subjected only to heat (‘Heat’) compared with untreated control (‘Control’) (Figure 1A–C). In contrast, plants pretreated with SA before experiencing heat stress (‘SA+Heat’) displayed a noticeable improvement in their phenotypic traits relative to the ‘Heat’ group (Figure 1A–C). Correspondingly, ‘Heat’ plants displayed a significant decrease in shoot height, shoot fresh weight (FW), shoot dry weight (DW), root FW, and root DW by 15.72%, 62.97%, 24.45%, 61.53%, and 20.20%, respectively, in relation to ‘Control’ plants (Figure 1D–H). Remarkably, SA pretreatment (‘SA+Heat’) significantly improved shoot height by 36.05%, shoot FW by 232.75%, shoot DW by 100.72%, root FW by 216.67%, and root DW by 63.29%, compared with the plants exposed solely to heat stress (‘Heat’) (Figure 1D–F). Additionally, plants that underwent SA pretreatment alone (‘SA’) showed significant improvements in shoot height by 15.58%, shoot FW by 35.72%, shoot DW by 75.27%, root FW by 42.30%, and root DW by 46.97%, in relation to ‘Control’ plants (Figure 1D–F).

### 2.2. Comparative Analysis of Photosynthetic Pigments and Flavonoids in Salicylic Acid-Treated and Heat-Stressed Cotton

**Figure 2:**
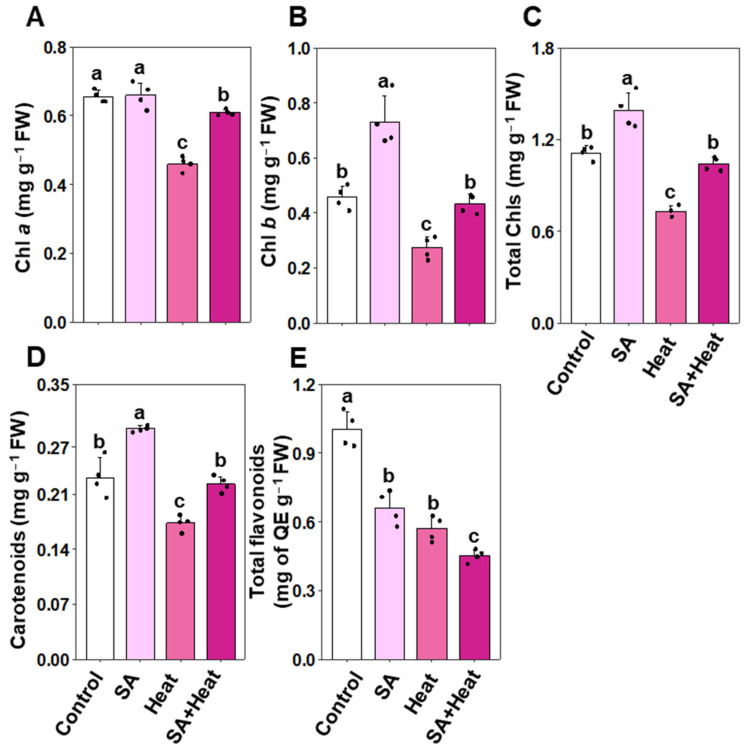
Impact of exogenously applied salicylic acid (SA, 1 mM) on the levels of photosynthetic pigments and flavonoids in cotton leaves subjected to 48 h of heat stress. (**A**) Chlorophyll *a* (Chl *a*), (**B**) chlorophyll *b* (Chl *b*), (**C**) total chlorophylls (total Chls), (**D**) carotenoids, and (**E**) total flavonoids in cotton leaves. Data are presented as mean values with standard deviations (*n* = 4). Statistically significant treatment differences, as assessed by the least significant difference (LSD) test (*p* < 0.05), are indicated by distinct letters above the bars. The treatments compared include water-treated non-stressed plants (Control), 1 mM SA-primed non-stressed plants (SA), water-treated heat-exposed plants (Heat), and 1 mM SA-primed heat-exposed plants (SA+Heat). Fresh weight (FW), quercetin equivalent (QE).

To thoroughly assess the impact of SA on the preservation of photosynthetic pigments and flavonoid levels under heat stress conditions, we conducted a detailed quantification of total chlorophylls, chlorophyll *a*, chlorophyll *b*, carotenoids, and total flavonoids (Figure 2A–E). Our analysis revealed that heat stress substantially reduced the levels of chlorophyll *a* by 29.83%, total chlorophylls by 34.16%, carotenoids by 25.09%, and total flavonoids by 43.25% in ‘Heat’ plants compared with the ‘Control’ plants. Interestingly, chlorophyll *b* levels remained relatively consistent between the ‘Heat’ and ‘Control’ plants (Figure 2A–E). Conversely, ‘SA+Heat’ plants exhibited a noteworthy increase in the quantities of chlorophyll *a* (by 32.37%), total chlorophylls (42.19%), and carotenoids (28.51%) in comparison to ‘Heat’ plants, while changes in leaf chlorophyll *b* and total flavonoid levels were not statistically significant (Figure 2A–E). ‘SA’ plants, on the other hand, displayed a substantial enhancement in chlorophyll *b* (by 59.96%), total chlorophylls (24.95%), and carotenoid levels (26.61%), compared with the ‘Control’ plants, albeit with a 34.15% reduction in total flavonoids (Figure 2B–E). Despite the treatment, the chlorophyll content in plants treated with salicylic acid (‘SA’) remained unchanged and was comparable to the levels found in the control plants (‘Control’), indicating that SA did not significantly alter chlorophyll concentration under non-stress conditions (Figure 2A).

### 2.3. Salicylic Acid Reduced Heat-Induced Oxidative Stress in Cotton Plants 

**Figure 3:**
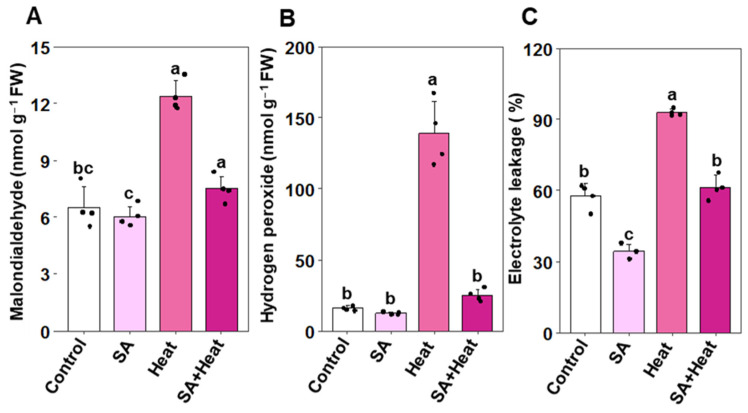
Impact of exogenously applied salicylic acid (SA, 1 mM) on oxidative stress markers in cotton leaves subjected to 48 h of heat stress. (**A**) Malondialdehyde content, (**B**) hydrogen peroxide content, and (**C**) electrolyte leakage percentage in cotton leaves. Data are presented as mean values with standard deviations (*n* = 4). Statistically significant treatment differences, as assessed by the least significant difference (LSD) test (*p* < 0.05), are indicated by distinct letters above the bars. Treatments include water-treated non-stressed plants (Control), 1 mM SA-primed non-stressed plants (SA), water-treated heat-exposed plants (Heat), and 1 mM SA-primed heat-exposed plants (SA+Heat).

To explore the potential of SA in alleviating heat-induced oxidative injury, we quantified MDA and H_2_O_2_ concentrations, along with the percentage of electrolyte leakage in cotton leaves (Figure 3A–C). Compared with ‘Control’, leaves from plants exposed to heat stress (‘Heat’) demonstrated a notable increase in MDA (by 89.69%) and H_2_O_2_ (772.40%), alongside a 61.44% rise in electrolyte leakage (Figure 3A–C). Remarkably, SA priming before heat stress (‘SA+Heat’) significantly reduced MDA levels by 39.49%, H_2_O_2_ levels by 82.06%, and electrolyte leakage by 33.99% in comparison to the ‘Heat’ group (Figure 3A–C). Furthermore, leaves from plants treated with SA alone (‘SA’) displayed a substantial reduction in electrolyte leakage by 40.36%, although the levels of MDA and H_2_O_2_ remained comparable, similar to the ‘Control’ group (Figure 3A–C).

### 2.4. Differential Antioxidant Enzyme Responses to Salicylic Acid and Heat Stress in Cotton

**Figure 4:**
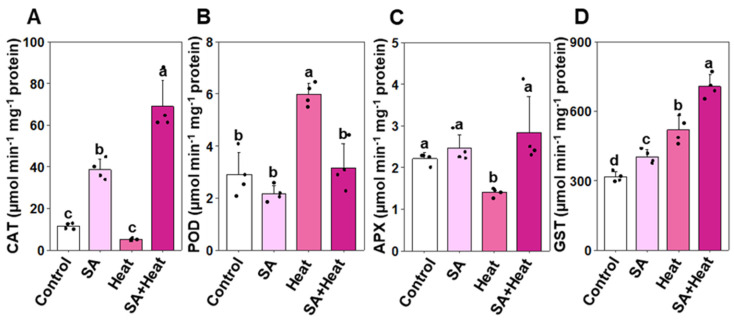
Impact of exogenously applied salicylic acid (SA, 1 mM) on the activities of key antioxidant enzymes in cotton leaves subjected to 48 h of heat stress. (**A**) Catalase (CAT), (**B**) peroxidase (POD), (**C**) ascorbate peroxidase (APX), and (**D**) glutathione *S*-transferase (GST) activities in cotton leaves. Data are presented as mean values with standard deviations (*n* = 4). Statistically significant treatment differences, as assessed by the least significant difference (LSD) test (*p* < 0.05), are indicated by distinct letters above the bars. Treatments include water-treated non-stressed plants (Control), 1 mM SA-primed non-stressed plants (SA), water-treated heat-exposed plants (Heat), and 1 mM SA-primed heat-exposed plants (SA+Heat).

To elucidate the effect of SA on enhancing antioxidant defense mechanisms, we evaluated the activities of several crucial antioxidant enzymes, namely GST, APX, POD, and CAT (Figure 4A–D). Heat stress substantially improved POD and GST activities by 106.61% and 64.09%, respectively, in ‘Heat’ plants compared with ‘Control’ plants. Conversely, APX activity was reduced by 36.69%, while CAT activity remained comparable in ‘Heat’ and ‘Control’ plants (Figure 4A–D). Remarkably, ‘SA+Heat’ plants showed a notable improvement in CAT (by 1243.08%), APX (102.66%), and GST (35.88%) activities, and a notable decrease in POD activity (46.99%) relative to ‘Heat’ plants (Figure 4A–D). Additionally, the activities of POD, APX, and GST in salicylic acid-treated (‘SA’) plants were comparable to those in control (‘Control’) plants. However, CAT activity was markedly higher, showing an increase of 237.14% in ‘SA’ plants compared with ‘Control’ plants (Figure 4A–D). 

### 2.5. Salicylic Acid’s Role in Enhancing Water Status and Osmoprotectant Balance in Cotton Plants under Heat Stress

**Figure 5:**
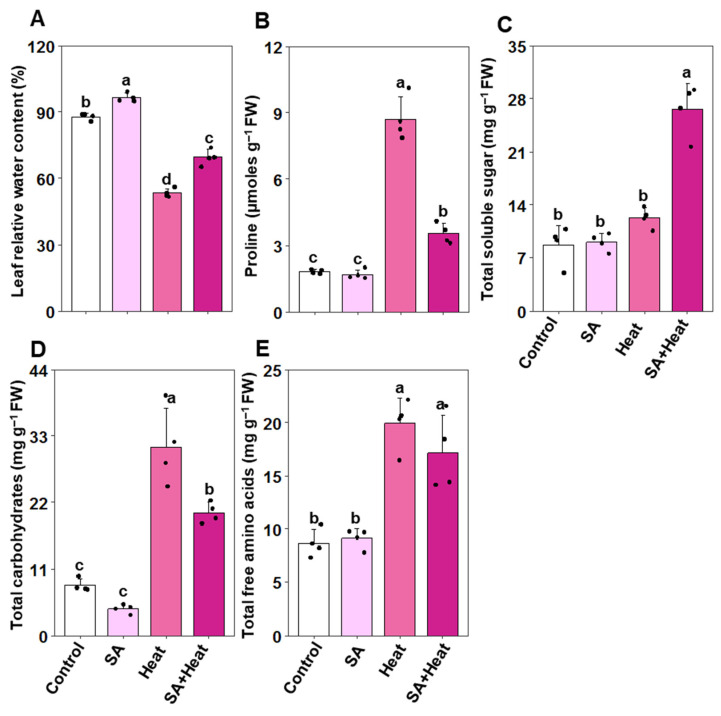
Impact of exogenously applied salicylic acid (SA, 1 mM) on various physiological and biochemical parameters in cotton leaves subjected to 48 h of heat stress. (**A**) Leaf relative water content, (**B**) proline, (**C**) total soluble sugars, (**D**) total carbohydrates, and (**E**) total free amino acid levels in cotton leaves. Data are presented as mean values with standard deviations (*n* = 4). Statistically significant treatment differences, as assessed by the least significant difference (LSD) test (*p* < 0.05), are indicated by distinct letters above the bars. Treatments include water-treated non-stressed plants (Control), 1 mM SA-primed non-stressed plants (SA), water-treated heat-exposed plants (Heat), and 1 mM SA-primed heat-exposed plants (SA+Heat). Fresh weight (FW).

To evaluate whether salicylic acid (SA) aids in maintaining water status under heat stress conditions, we measured the relative water content (RWC) and analyzed the concentrations of total soluble sugars, proline, total carbohydrates, and total free amino acids in the leaves of cotton plants (Figure 5A–E). Plants exposed to heat stress (‘Heat’) experienced a substantial reduction in RWC by 39.52% compared with control plants (‘Control’). Additionally, these heat-stressed plants exhibited significant increases in proline levels (378.08%), total carbohydrates (275.29%), and total free amino acids (130.22%). In contrast, the levels of total soluble sugars remained unchanged when compared with the ‘Control’ group (Figure 5A–E). Conversely, SA-primed (‘SA+Heat’) plants showed a notable decrease in proline levels (by 59.36%) and total carbohydrates (34.90%), alongside improvement in RWC (30.59%) and total soluble sugars (116.30%) relative to ‘Heat’ plants (Figure 5A–D). However, there was no significant difference in the total free amino acid content between the ‘SA+Heat’ plants and those subjected to ‘Heat’ treatment alone (Figure 5E). Compared with the ‘Control’ plants, those treated with salicylic acid (‘SA’) demonstrated a significant enhancement in leaf RWC, showing an increase of 9.59%. However, the levels of osmoprotectants remained largely unchanged between the ‘Control’ and ‘SA’ plants (Figure 5A–E). 

### 2.6. Visualizing the Differential Impact of Heat and Salicylic Acid Treatments on Cotton Plants: A Detailed Heatmap and Principal Component Analysis Approach

**Figure 6:**
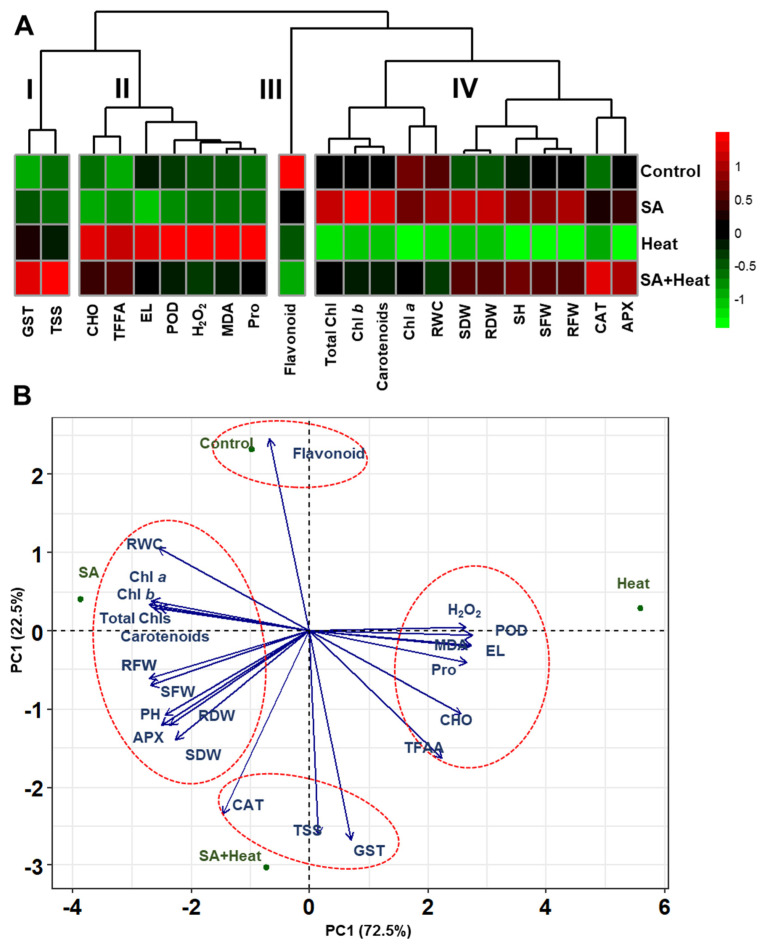
Clustering heatmap and biplot principal component analysis (PCA) illustrating the impact of salicylic acid (SA) priming on heat stress tolerance in cotton plants. (**A**) Clustering heatmap of normalized mean values showing the responses of various morphophysiological and biochemical parameters under different treatment combinations: water-treated non-stressed plants (Control), 1 mM SA-primed non-stressed plants (SA), water-treated heat-exposed plants (Heat), and 1 mM SA-primed heat-exposed plants (SA+Heat). The heatmap provides a comprehensive overview of the divergence in parameter responses across treatments. (**B**) Biplot PCA depicts correlations between parameters and treatments, with the first two principal components (PC1 and PC2) explaining 94.6% of the total variability in the dataset. Vector lines indicate the strength and direction of relationships between morphophysiological and biochemical parameters and treatments, with tiny angles denoting weak associations and wide angles indicating strong associations. Key parameters include plant height (PH), chlorophyll *a* (Chl *a*), shoot dry weight (SDW), chlorophyll *b* (Chl *b*), shoot fresh weight (SFW), root dry weight (RDW), root fresh weight (RFW), total chlorophylls (Total Chls), ascorbate peroxidase (APX), total free amino acids (TFAA), peroxidase (POD), total flavonoids (Flavonoid), glutathione *S*-transferase (GST), malondialdehyde (MDA), total carbohydrates (CHO), hydrogen peroxide (H_2_O_2_), electrolyte leakage (EL), proline (Pro), relative water content (RWC), and total soluble sugars (TSS).

To succinctly visualize the impact of different treatment compositions on cotton plants at a glance, we generated a heatmap that delineated four distinct clusters of parameters (Figure 6A). In ‘Heat’ plants, parameters within the cluster-I displayed an increasing trend compared with ‘Control’ plants, with an unexpected further increase observed in ‘SA+Heat’ plants (Figure 6A). Conversely, variables in cluster-II increased significantly in ‘Heat’ plants relative to ‘Control’ plants but demonstrated a decrease in ‘SA+Heat’ plants (Figure 6A). Notably, the parameter in cluster-III uniquely exhibited an increasing trend in ‘Control’ plants compared to all other treatments (Figure 6A). In contrast, the cluster-IV variables showed a decrease in the ‘Heat’ plants but exhibited an increase in the ‘SA+Heat’ plants compared with the ‘Heat’ plants (Figure 6A). Subsequently, we utilized principal component analysis (PCA) to investigate the relationships between the various treatments and the measured parameters. This analytical approach allowed us to discern patterns and correlations within the data, providing deeper insights into the effects of different treatments on the observed variables (Figure 6B). The analysis revealed that the majority of variability was explained by PC1 (72.5%) and PC2 (22.50%), cumulatively accounting for 95.00% of the observed variability. Intriguingly, cluster-I parameters were strongly associated with the ‘SA+Heat’ treatment, while cluster-II variables correlated closely with the ‘Heat’ treatments (Figure 6A,B). ‘Control’ plants predominantly aligned with the cluster-III parameter (Figure 6A,B), whereas variables from cluster-IV were closely linked to the ‘SA’ treatment (Figure 6A,B).

## 3. Discussion

Global cotton growth and production are severely perturbed by high-temperature stress, a challenge increasingly prevalent in many regions worldwide [34]. SA has long been deemed as an unwavering warrior, safeguarding plants from the pernicious consequences imposed by a multitude of abiotic stresses in a diverse array of crop species, including rice (*Oryza sativa*), cotton, maize (*Zea mays*), and wheat (*Triticum aestivum*) [25,35,36,37]. Our study demonstrated that the supplementation of SA markedly enhances the heat tolerance of cotton plants. This conclusion is supported by SA’s significant role in mitigating heat-induced phenotypic abnormalities, including leaf wilting and shrinkage. The observed reduction in these stress symptoms is likely correlated with the notable improvements in overall growth and biomass yield in SA-treated plants (Figure 1A–F). It is well-recognized that under high-temperature stress, plants need to preserve root plasticity to efficiently explore the surrounding and deeper layers of soil to extract the water to cool the plant’s overall temperature [19,38,39,40]. Therefore, robust root growth under elevated temperatures is one of the paramount traits that enhances the heat tolerance of plants. Our investigation displayed that the root biomass of heat-stressed plants was substantially reduced; nonetheless, SA supplementation successfully promoted robust root growth and amplified biomass (Figure 1F). This finding indirectly suggests that the profound root system in SA-supplemented plants enabled them to eagerly absorb copious amounts of water from the soil, ultimately resulting in vigorous growth under high-temperature conditions. In support of our investigation in enhancing plant growth and biomass, we concurrently observed that the supplementation of SA enhanced photosynthetic pigment levels, such as chlorophyll *a*, chlorophyll *b*, total chlorophylls, and carotenoids, in cotton leaves subjected to both non-stressed and heat-stressed conditions (Figure 2A–D). These outcomes indicate that SA may be involved in either stimulating the production or inhibiting the breakdown of photosynthetic pigments, or perhaps both, consequently leading to an enhancement in biomass production (Figure 2A–D). Our PCA analysis also revealed a positive correlation with SA-treated plants in growth features and photosynthetic pigment contents (Figure 6A,B).

A substantial body of research has consistently demonstrated that the decrease in biomass observed under heat stress conditions is intricately connected to the initiation of oxidative damage. This damage is predominantly driven by the elevated production of ROS, which disrupts cellular homeostasis and impairs various physiological processes critical for plant growth and development [41,42,43,44]. In the present investigation, we observed substantially higher levels of EL percentage, MDA, and H_2_O_2_ in heat-exposed cotton plants, unequivocally demonstrating the profound impact of heat stress on inducing severe oxidative stress and membrane dysfunction in cotton plants (Figure 3A–C). Furthermore, SA priming followed by heat stress exposure led to a reduced accumulation of H_2_O_2_, along with lower levels of MDA and EL. This indicates that SA effectively mitigates cell membrane damage by preventing the excessive production of H_2_O_2_ and curtailing lipid peroxidation product (MDA) in heat-stressed leaves. The findings of our PCA also indicate that oxidative stress markers, including H_2_O_2_ and MDA, were intricately associated with ‘Heat’ treatment (Figure 6A,B). This observation aligns with recent studies, which have shown that SA can mitigate oxidative damage in cotton leaves under salt stress and in tomato (*Solanum lycopersicum*) under heat stress, as well as plays a role in regulating leaf senescence and promoting shoot growth [26,45]. Furthermore, our findings unequivocally displayed that those plants supplemented with SA exhibited a robust antioxidant defense system that effectively mitigated the oxidative burden imposed by H_2_O_2_ and consequential effects derived from EL and lipid peroxidation under heat stress conditions. We observed that cotton plants primed with SA and subsequently exposed to heat stress triggered a momentous upsurge in the activities of CAT, APX, and GST, which played a pivotal role in efficiently detoxifying the heat-stress-induced H_2_O_2_, ensuring plant survival and resilience under such challenging conditions (Figure 3B and Figure 4A,C,D). Similar to our findings, Janda et al. [46] also revealed that SA treatment bolstered GST activity in *Brachypodium distachyon* plants under heat-stress conditions. Our PCA also revealed a strong association in heightened levels of antioxidant enzyme activities with the ‘SA+Heat’ treatment (Figure 6A,B).

Plants facing stressful conditions encounter a formidable challenge as their water uptake capacity diminishes, resulting in a disruptive balance of water at the cellular level [47]. In response, an arsenal of osmoprotectants, such as proline, is mobilized to mitigate this imbalance [25,48]. Our study revealed that cotton plants subjected to heat stress exhibited escalated proline levels while concurrently experiencing significantly diminished leaf RWC in comparison to control plants (Figure 5A,B). These compelling outcomes suggest that proline accumulation alone may not suffice to maintain optimal water status under heat stress. Intriguingly, SA priming effectively minimized water loss in heat-stressed cotton plants without significantly increasing proline levels, as opposed to the traditional view that proline accumulation directly correlates with stress severity and suggested alternative mechanisms for preserving leaf RWC under heat stress [25,48,49,50]. Remarkably, we also noted a surge in total free amino acid levels in heat-exposed plants, in contrast to SA-primed heat-stressed plants; nonetheless, even this surge proved inadequate in sustaining the plant’s water status (Figure 5E). Additionally, we observed that heat-stressed plants treated with SA exhibited a significantly higher accumulation of total soluble sugars compared to those treated with water. This increased sugar accumulation likely plays a crucial role in maintaining plant water status, as indicated by the higher RWC in the leaves (Figure 5A,C). However, we observed a pronounced reduction in RWC in heat-stressed plants. Apart from heat stress, this sharp decline is likely attributable to insufficient irrigation intended to mitigate water stress, alongside minor yet significant variations in relative humidity inside the growth chamber. These factors necessitate meticulous monitoring and control in the design and execution of heat stress experiments. Furthermore, scientific evidence explicitly highlights the profound impact of amplified total soluble sugar accumulation in meeting the crucial nitrogen and carbon demands to maintain optimal plant metabolic functions under stress circumstances [51]. Remarkably, Zhang et al. [52] have also extensively reported the protective role of heightened total soluble sugars in counteracting the adverse effects induced by heat stress in rice plants. In addition, the reduced carbohydrate content in SA-primed cotton plants may enhance osmotic regulation by providing additional sugars for osmoprotection. This balance could be achieved through the interconversion of starch to sugars via hydrolytic enzymes and/or by supplying ATP to energize stressed cells through an elevated respiration rate (Figure 5C,D) [53,54].

## 4. Materials and Methods

### 4.1. Plant Species and Treatment Compositions

CB-12 cotton seeds, renowned for their high yield potential in Bangladesh (seed cotton yield: 4.52 tons ha^−1^), were pre-soaked overnight in darkness before being sown in polybags (19 cm in length and 15 cm in width) containing 800 g of soil. These polybags were subsequently placed in a growth chamber (GC-560H, Firstek Scientific, Taiwan), where conditions were carefully controlled to simulate extended daylight. The chamber maintained a 16-h day and an 8-h night photoperiod, with the temperature set at 28 °C, relative humidity at 70%, and lighting at 500 μmol m^−2^ s^−1^ lights. Once uniformly germinated, the seedlings (four per polybag) were categorized into two main groups. One group received treatment with SA (Fujifilm Wako Pure Chemical Corporation, Osaka, Japan), while the control group was treated with tap water. 

SA treatments were administered by applying 10 mL of a 1 mM SA solution directly to the root zone of each plant, totaling 40 mL for four seedlings in each polybag. This was performed at two developmental stages, seven and twelve days after germination, based on the optimal dose identified in a previous study conducted by Keya et al. [25]. Control plants received an equal volume of tap water. Seventeen days post-germination, which includes four days following the last SA and tap water application, plants were subdivided into four groups, each consisting of five polybags to serve as five replications. These groups were then subjected to different treatments: (i) water-treated, non-stressed plants (Control), (ii) 1 mM SA-primed, non-stressed plants (SA), (iii) water-treated, heat-exposed plants (Heat), and (iv) 1 mM SA-primed, heat-exposed plants (SA+Heat). Heat stress was induced by exposing the plants to 42 °C temperature and 70% humidity for 48 h. To mitigate water stress, 30 mL of water was added to each of the heat-stressed polybags at 12-h intervals, totaling 4 times applications over the stress tenure. To ensure its reliability, research was conducted three times. The 3rd leaves, counting from the base, of 19-day-old plants were collected for the analysis of various morphological, physiological, and biochemical parameters.

### 4.2. Growth Parameter Assessment

Height, shoot dry weight (DW), and root biomass were assessed from three plants randomly selected from each treatment group, following the comprehensive methodologies described by Rahman et al. [47].

### 4.3. Methodical Approach to Evaluating Water Content and Cellular Integrity in Cotton Leaves

To measure the relative water content (RWC) of cotton leaves, we followed a systematic procedure. First, we weighed the fresh leaves (FW). Then, the leaves were soaked in deionized water for twelve hours to achieve full turgidity, after which they were reweighed to obtain the turgid weight (TW). The leaves were subsequently dried at 75 °C for 72 h to determine their dry weight (DW) [51]. The RWC was calculated using the formula: RWC (%) = [(FW − DW)/(TW − DW)] × 100. To determine the electrolyte leakage (EL), we adopted a specific method. We submerged 0.2 g of leaf tissue in 20 milliliters of tap water. The initial electrical conductivity of the solution (EC1) was measured. After heating the samples at 100 °C for thirty minutes, the electrical conductivity was measured again (EC2) after cooling the sample. The EL was then calculated using the formula EL (%) = [(EC1 − EC0)/(EC2 − EC0)] × 100, with EC0 representing the conductivity of the tap water [48].

### 4.4. Assessment of Photosynthetic Pigments and Flavonoid Contents

Pigments were extracted from 0.2 g of freshly harvested cotton leaves using chilled 80% acetone (*v*/*v*). The suspension was centrifuged at 10,000× *g* for 10 min at 4 °C. The concentrations of the supernatant were then quantified using a spectrophotometer. For carotenoids, the quantification was based on equations from Lichtenthaler and Wellburn [55], while chlorophyll *a*, chlorophyll *b*, and total chlorophyll levels were measured using methods established by Arnon [56]. Furthermore, we evaluated the total flavonoid content in fresh leaf samples using the detailed protocol described by Das et al. [48].

### 4.5. Assessment of Hydrogen Peroxide, Malondialdehyde, and Proline

To quantify the biochemical markers indicative of oxidative stress, we utilized the 3rd bottommost leaves from freshly harvested cotton plants. Spectrophotometric methods, as detailed by Kim et al. [57] for malondialdehyde (MDA) determination, were employed. Briefly, leaf samples (0.2 g) were homogenized in 800 µL of 0.1% trichloroacetic acid (TCA). The homogenate underwent centrifugation at 14,000× *g* for 20 min at 4 °C to isolate the supernatant. Subsequently, 500 µL of the supernatant was reacted with 1.5 mL of 0.5% thiobarbituric acid in 20% TCA. The reaction mixture was then heated to 95 °C for 30 min. Following incubation, the mixture was promptly cooled on ice and centrifuged at 10,000× *g* for 5 min. The absorbance of the resulting supernatant was measured at 532 nm and 600 nm using a GENESYS 10S UV–VIS spectrophotometer (Thermo Scientific, San Jose, CA, USA). MDA concentrations were precisely calculated using an extinction coefficient of 156 mM^−1^ cm^−1^. For the determination of hydrogen peroxide (H_2_O_2_), fresh plant tissues (0.1 g) were pulverized in 2 mL of 0.1% (*w*/*v*) trichloroacetic acid (TCA) under pre-chilled conditions using an ice bath. The homogenate was then subjected to centrifugation at 12,000× *g* for 15 min. The supernatant (0.5 mL) was subsequently aliquoted and mixed with 0.5 mL of potassium phosphate buffer (pH 7.0) and 1 mL of potassium iodide solution. The resultant mixture was vigorously vortexed, and the absorbance was quantitatively measured at 390 nm, with 0.1% TCA serving as the blank [58]. Additionally, to evaluate the proline content, which serves as a vital osmoprotectant and stress biomarker in plants, we employed the protocol established by Bates et al. [59]. 

### 4.6. Extraction of Antioxidant Enzymes and Quantification of Enzyme Activities

Following the methodology detailed by Rahman et al. [47], we extracted enzymes from cotton leaf samples to evaluate the activities of several key antioxidant enzymes. These enzymes included glutathione *S*-transferase (GST), peroxidase (POD), catalase (CAT), and ascorbate peroxidase (APX).

### 4.7. Quantification Techniques for Soluble Sugars, Amino Acids, and Carbohydrate Levels

To conduct biochemical analyses, we carefully excised the third leaf from the base of each plant. The quantification of soluble sugar levels was conducted using Somogyi’s method [60]. Briefly, an extraction buffer was prepared by mixing 80 mL of ethanol with 20 mL of water. For the anthrone reagent, dissolve anthrone powder in sulfuric acid (2.5 mL acid: 5 mg anthrone) on ice, stirring until completely dissolved. Standard glucose stock was prepared by dissolving 20 mg of glucose in 20 mL of water (1000 µg/mL) and then diluting accordingly. Homogenize 0.2 g of fresh leaf sample in 1.5 mL of 80% ethanol, centrifuge at 11,500× *g* for 15 min at 4 °C and collect the supernatant. Dilute 40 µL of the supernatant with 160 µL of water. Slowly add 2.5 mL of the anthrone reagent to the mixture, then vortex and incubate at 90 °C for 15 min. Cool immediately on ice, then measure the absorbance at 620 nm using the anthrone reagent as the blank. Free amino acids were measured following the technique described by Lee and Takahashi [61]. An extraction buffer was prepared by mixing 80 mL of ethanol with 20 mL of distilled water. For the reaction mixture (RM), combine citrate buffer (0.5 M, pH 5.5), 1% ninhydrin reagent (prepared by dissolving ninhydrin in citrate buffer), and 55% glycerol solution, stirring with a magnetic stirrer. For each sample, 0.2 mL of 1% ninhydrin reagent, 0.2 mL of citrate buffer, and 1.2 mL of 55% glycerol are required to prepare the RM. Homogenize 0.2 g of fresh leaf sample in 1 mL of 80% ethanol. Centrifuge the mixture at 11,500× *g* at 4 °C for 15 min and collect the supernatant. Mix 0.1 mL of the supernatant with 1.6 mL of RM. For the blank, mix 0.1 mL of 80% ethanol with 1.6 mL of RM. Vortex and heat the mixture in a boiling water bath for 30 min until a purple color develops. After cooling, measure the absorbance at 570 nm using a spectrophotometer. Additionally, we determined total carbohydrate content using the protocol established by Dubois et al. [62].

### 4.8. Statistical Methodologies and Visualization Tools for Data Interpretation

The data were statistically analyzed using Statistix 10 software (Analytical Software, 2105 Miller Landing Rd, Tallahassee, FL 32312, USA). We applied a one-way analysis of variance (ANOVA) to evaluate differences across all datasets, and significant variations between treatments were identified using the least significant difference (LSD) post hoc test with a significance level of *p* < 0.05. The results, presented as means ± standard deviations (SDs), were based on three biological replicates for morphological data and four biological replicates for biochemical data per treatment. To further explore the data, we employed cluster heatmap and principal component analysis (PCA) techniques, utilizing various R software packages (RStudio version 2023.06.1+524). This approach followed the methodology outlined by Ghosh et al. [63], enabling us to visualize complex patterns and relationships within the dataset effectively.

## 5. Conclusions

**Figure 7:**
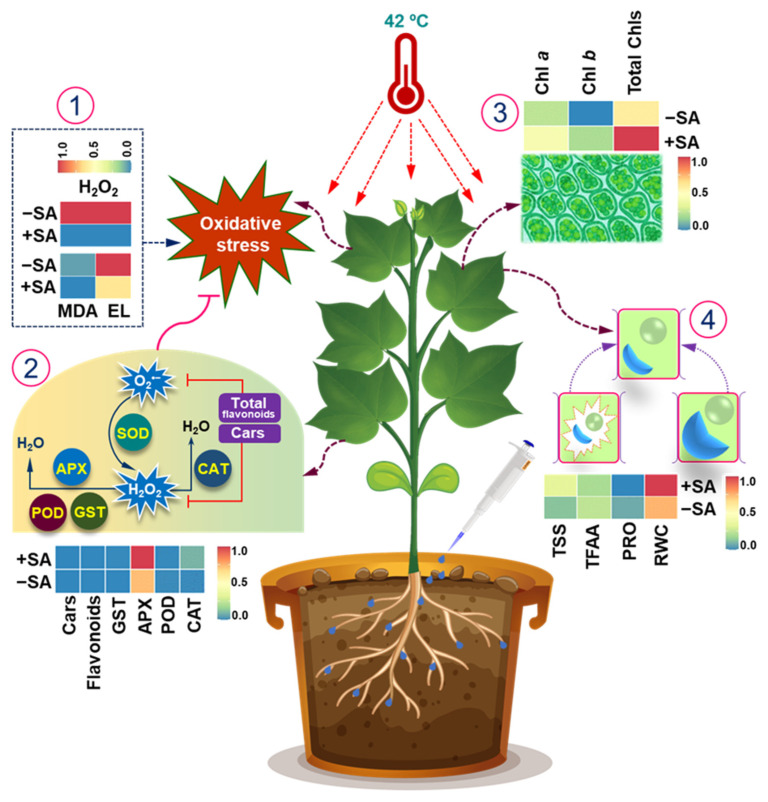
Mechanistic overview of salicylic acid (SA) priming in enhancing heat stress tolerance in cotton plants. SA priming significantly reduces the levels of oxidative stress indicators such as malondialdehyde (MDA), which is a marker of lipid peroxidation, electrolyte leakage (EL), which indicates cell membrane damage, and hydrogen peroxide (H_2_O_2_), a reactive oxygen species. Concurrently, SA priming enhances the activities of several key antioxidant enzymes that play vital roles in mitigating oxidative damage. These enzymes include glutathione *S*-transferase (GST), which is involved in detoxification processes; ascorbate peroxidase (APX), which helps in the reduction of H_2_O_2_ to water; catalase (CAT), which decomposes H_2_O_2_ into water and oxygen; and peroxidase (POD), which catalyzes the oxidation of various substrates using H_2_O_2_ (1, 2). This robust antioxidant defense mechanism alleviates oxidative damage, thereby preserving photosynthetic efficiency, as evidenced by higher levels of photosynthetic pigments (chlorophyll *a*, chlorophyll *b*, and Cars) (3). Additionally, SA-primed plants demonstrate a superior ability to maintain higher relative water content (RWC), which is crucial for sustaining cellular functions and overall plant turgor under stress conditions. These plants also accumulate significantly greater amounts of total soluble sugars (TSS), which act as osmoprotectants and energy sources, aiding in stress mitigation. The levels of proline (PRO), an amino acid known for its role in osmotic adjustment, protection of cellular structures, and scavenging of free radicals, are also elevated. Furthermore, SA priming leads to an increase in total free amino acids (TFAA), which contribute to protein synthesis, stress signaling, and metabolic adjustments during heat stress (4). The depicted interactions underscore the multifaceted role of SA in modulating physiological and biochemical responses to heat stress in cotton plants. Cars, carotenoids.

Our findings demonstrated that salicylic acid (SA) significantly enhances cotton growth under optimal conditions and improves its resilience against heat stress (Figure 7). This underscores SA pretreatment’s potential as a novel strategy to increase heat tolerance in crops. Specifically, SA pretreatment led to notable improvements in key heat tolerance mechanisms, including increased shoot and root biomass, preservation of essential photosynthetic pigments, effective reduction of H_2_O_2_, EL, and MDA-mediated oxidative damage through the strengthening of the antioxidant defense system, and a remarkable increase in osmoprotectant levels to maintain water balance (Figure 7). Future research should aim to decipher the specific regulatory pathways that SA targets and modulates to impart heat tolerance in cotton and other essential fiber crops through comprehensive genetic and molecular investigations. Additionally, conducting field trials and economic assessments of SA treatments are critical next steps to mitigate heat stress impacts and minimize catastrophic losses in crop yields.

## Data Availability

Data will be available upon request.
